# Determinants of Pain-Induced Disability in German Women with Endometriosis during the COVID-19 Pandemic

**DOI:** 10.3390/ijerph19148277

**Published:** 2022-07-06

**Authors:** Roxana Schwab, Kathrin Stewen, Tanja Kottmann, Susanne Theis, Tania Elger, Bashar Haj Hamoud, Mona W. Schmidt, Katharina Anic, Walburgis Brenner, Annette Hasenburg

**Affiliations:** 1 Department of Obstetrics and Gynecology, University Medical Center of the Johannes Gutenberg University Mainz, Langenbeckstr. 1, 55131 Mainz, Germany; kathrin.stewen@unimedizin-mainz.de (K.S.); susanne.theis2@unimedizin-mainz.de (S.T.); tania.elger@unimedizin-mainz.de (T.E.); mona.schmidt@unimedizin-mainz.de (M.W.S.); katharina.anic@unimedizin-mainz.de (K.A.); walburgis.brenner@unimedizin-mainz.de (W.B.); annette.hasenburg@unimedizin-mainz.de (A.H.); 2 CRO Dr. med. Kottmann, 59077 Hamm, Germany; tk@cro-kottmann.de; 3 Department for Gynecology, Obstetrics and Reproductive Medicine, Saarland University Hospital, 66424 Homburg, Germany; bashar.hajhamoud@uks.eu

**Keywords:** pain-induced disability, chronic pain, endometriosis, mental health, resilience, social support, COVID-19

## Abstract

(1) Background: The main aim of this research was to examine the factors leading to pain-induced disability by assessing the impact of demographic, endometriosis-specific, pandemic-specific, and mental health factors. (2) Methods: Women with endometriosis who attended online support groups were invited to respond to an online survey during the first wave of the COVID-19 pandemic in Germany. The Pain Disability Index (PDI) was employed to assess disability-related daily functioning. Independent predictors of pain-induced disability were determined using univariate and multivariate logistic regression analyses. (3) Results: The mean PDI score of the study population was 31.61 (SD = 15.82), which was significantly higher (*p* < 0.001) than that reported in a previously published normative study of the German population. In the present study, a high level of pain-induced disability, as defined by scores equal to or higher than the median of the study population, older age (OR 1.063, 95% CI 1.010–1.120, *p* = 0.020), dysmenorrhea (OR 1.015, 95% CI 1.005–1.026, *p* = 0.005), dysuria (OR 1.014; 95% CI 1.001–1.027, *p* = 0.029), lower back pain (OR 1.018, 95% CI 1.007–1.029, *p* = 0.001), and impaired mental health (OR 1.271, 95% CI 1.134–1.425, *p* < 0.001) were found to be independent risk factors. Pandemic-specific factors did not significantly influence the pain-induced disability of the participants in this study. (4) Conclusions: The level of pain-induced disability was significantly higher among the women with endometriosis than among women in the normative German validation study. Our findings identified risk factors for experiencing a high level of pain-induced disability, such as demographic and specific pain characteristics. Pandemic-specific factors did not significantly and independently influence the pain-induced disability during the first wave of the COVID-19 pandemic in Germany. Impaired mental health negatively influenced functioning during daily activities. Thus, women with endometriosis should be managed by a multidisciplinary team of healthcare professionals to prevent negative effects of pain-induced disability on their quality of life.

## 1. Introduction

Endometriosis is a common chronic gynecologic inflammatory disease caused by the growth of endometrial-like tissue outside the uterine cavity [1,2]. The community prevalence of endometriosis is estimated to be about 10–15% of women of reproductive age, but the condition may affect adolescents and very young girls as well [1,2]. Endometriosis is present in up to 30–50% of women with pain and/or infertility [3], resulting in approximately 176 million affected women worldwide [2,4].

An induced chronic, inflammatory reaction of the abdominal cavity to endometriotic lesions leads to various nonspecific symptoms, such as diarrhea, constipation, fatigue, flatulence, and/or infertility [2]. Pain is a key symptom of the disease and may persist even after medical treatment and/or surgery. Women with endometriosis often experience symptoms such as dysmenorrhea, dyspareunia, dyschezia, or noncyclic pelvic pain [1,5,6,7]. Dysmenorrhea is experienced by over 75% of women with ovarian endometriosis or endometriosis of the peritoneum, non-menstrual pain is reported by 67%, and dyspareunia is reported by up to 50% of women with a subsequent diagnosis of endometriosis [8]. No direct relationship has been established between pain intensity and endometriosis severity or stage of disease [8]. Compared to the controls in one study without endometriosis but with pelvic pain, the women with endometriosis experienced higher levels of dyspareunia, chronic pain, and dysmenorrhea [9].

Pain intensity is one of the key factors in recommendations for the surgical confirmation of a definitive diagnosis of endometriosis. To date, no clear cut-off points for pain levels have been established for use in decisions regarding invasive procedures using laparoscopy in women with chronic or recurrent pelvic pain. Healthcare providers balance potential surgical risks on one hand and possible short- or long-term impairment of quality of life on the other hand. Up to 25% of women with pain associated with endometriosis do not benefit directly from surgery, as reported in a study in which pain symptoms were persistent, unchanged, or even worse after surgery for removal of endometriosis lesions [10,11].

Chronic pain is defined by the International Association for the Study of Pain (IASP) as an unpleasant sensation that persists for longer than three months [12]. As a result of pain and a substantial diagnostic delay of up to 10.4 years in Germany [7,13], women with endometriosis meet the IASP criteria for chronic pain. Chronic pain may lead to peripheral and central sensitization caused by increased neuro-excitability of the peripheral nociceptors, changes in gene expression of the central nervous system, and facilitation of the formation of pain memory [14]. As a result of their chronic pain, women with endometriosis experience a significant increase in physical and mental disabilities, and a decrease in their quality of life compared with that of normative populations [2,15,16,17,18,19,20]. Moreover, correlations between central sensitization and pain-induced disability have been reported in various chronic pain conditions, such as chronic spinal pain, musculoskeletal pain, and chronic pain in patients with cancer [21,22,23,24].

Chronic pain is not only an extended unpleasant physical experience, it is a complex biosocial phenomenon [25]. Disability is an important aspect of chronic pain conditions and reflects the burden of disease on each affected individual. Pain-induced disability is defined as the extent to which chronic pain interferes with daily functioning and the ability to participate in different life activities, such as family, recreational and work-related activities [26,27]. There seems to be a complex interplay between pain-induced disability and other pain-related factors, such as pain intensity and duration [27]. A recent study reported age and pain duration as independent predictors of disability in women with endometriosis [28]. Other predictive factors of pain-induced physical impairment, such as older age and pain severity, have been examined previously [29]. The level of pain-induced disability, but not the pain level itself, has been linked to increased morbidity and even mortality in patients with chronic pain [30]. Furthermore, a relationship between pain and mental health has been reported in studies of patients with chronic pain [13,25,31], and other studies have found that psychological distress and the perception of pain influence pain intensity, tolerability, and thus, pain-induced disability [25,32,33].

At the end of 2019, the novel severe acute respiratory syndrome coronavirus 2 (SARS-CoV-2) was first reported in China, and subsequently, coronavirus disease 2019 (COVID-19) spread rapidly among other countries by the beginning of 2020. On 30 January 2020, the World Health Organization declared the outbreak a “public health emergency of international concern”, and on 11 March 2020, COVID-19 met the criteria for a pandemic [34]. Public health measures, such as social distancing, quarantine, and locking down the economy, were implemented in various countries to prevent further spread [35], leading to a higher risk for social disconnection and loneliness. The COVID-19 pandemic also had a negative impact on vulnerable subgroups of the population. Women with endometriosis showed an increase in social and emotional vulnerability during the COVID-19 pandemic [13]. A study conducted in the Netherlands reported an increase in endometriosis-related complaints by more than 80% of women with endometriosis during the pandemic [36]. Similar results were found in a group of Spanish women with endometriosis [37], and a cross-sectional international study conducted by Ashkenazi et al. reported aggravation of symptoms in 35% of study participants [38]. As a result of the pandemic, a significant increase in mental health concerns, such as anxiety, depression, and lower resilience were observed in study populations worldwide [39,40,41,42,43]. Women diagnosed with endometriosis were at a high risk for developing mental health disorders, and their burden of psychological symptoms was greater with respect to mental health than the burden of the normative populations [19,36,38,44].

The factors leading to pain-induced disability in women with endometriosis are not sufficiently understood. This study investigated the relationship between sociodemographic, disease-specific, pandemic-specific, mental health, and resilience variables and self-reported high functional disability in various life domains among women with endometriosis in the context of the COVID-19 pandemic. We aimed to understand the underlying mechanisms of high functional pain-related disability to recognize women at risk for increased disability to prevent negative outcomes related to pain-induced disability (e.g., loss of professional or social opportunities, experiencing adverse psychological effects, or developing central pain sensitization). The results of this study were intended to help clinicians and patients equally to understand the impact of pelvic pain on women’s lives and to determine the optimal diagnostic and therapeutic procedures for each individual at risk for short- or long-term pain-related sequalae.

## 2. Materials and Methods

### 2.1. Recruitment of the Sample Population/Study Participants

Between 6 and 27 April 2020, an online questionnaire was activated on the Facebook internet platforms of patient support groups for German women diagnosed with endometriosis. Data collection was carried out anonymously using SoSciSurvey (a platform that stores all data in Germany and is subject to strict EU data protection laws). No personal information, such as the IP address, was collected. The participation in the study was voluntary. Inclusion criteria were age older than 18 years, a diagnosis of endometriosis during a surgical procedure, and informed consent to participate. 

The survey included questions related to the participants’ demographics (age, marital status, living alone, and educational level), disease-related (time since the diagnosis of endometriosis, age at diagnosis, delay of the endometriosis diagnosis, pain characteristics, pain intensity, and pain-induced disability), pandemic-related parameters (duration of a reduced social network, being in isolation or quarantine, and level of reduced social contacts), and mental health.

### 2.2. Pain Intensity

Pain intensity was assessed using a visual analog scale (VAS), a continuous scale with scores ranging from 0 (no pain) to 100 (worst pain imaginable) [45]. Participants were asked to complete a questionnaire on the intensity of their current pain (VAS_C_) and to report the intensity of their pain prior to the implementation of social distancing measures (VAS_P_). Current pain intensity was first evaluated as a continuous variable (VAS_C_) in order to conduct further analyses. In the second step, the intensity of current pain was divided into three categories: “no pain to low pain” VAS_CL_ (VAS = 0–44), “moderate pain” VAS_CM_ (VAS = 45–74), and “severe pain” VAS_PS_, VAS_CS_ (VAS = 75–100) [45].

### 2.3. Pain Disability Index (PDI)

The PDI rates pain-related disability in seven areas of daily life (family/home responsibilities, recreation, social activity, occupation, sexual behavior, self-care, and life-support activities). The items were scored on a scale from 0 (no interference) to 10 (total interference). Basic activities (the sum of self-care and life-support activities) and discretional activities (family/home responsibilities, recreation, social activity, occupation, and sexual behavior) were calculated to create sub-scores. Global pain-induced disability, which represented the total score of all the items, ranged from 0 to 70 [46].

### 2.4. Patient Health Questionnaire for Depression and Anxiety (PHQ-4)

The four-item PHQ-4 was used to assess the participants’ psychological burden. Two of the four PHQ items measure depression (PHQ-2), and the other two items measure generalized anxiety disorder (GAD-2) [47]. The reliability of the German version of the PHQ-4 is α = 0.78 [47].

Participants responded to the questions using a 4-point Likert-scale: “not at all” = 0, “several days but less than one week” = 1, “more than half the days” = 2, and “nearly every day” = 3. The PHQ-2, GAD-2, and PHQ-4 scores were calculated by adding the scores of the individual items [48,49,50,51]. A score ≥ 3 on the PHQ-2 or GAD-2 was considered a cut-off point between the normal range and the probable cases of major depression or generalized anxiety disorder (a “yellow flag” was used to indicate a high probability of a depressive or anxiety disorder, as these scores were higher than 93% of those of the normative general population). A PHQ-2 score ≥ 3 has a sensitivity of 82.9% and a specificity of 90.0% for predicting a major depressive disorder [52], and a GAD-2 ≥ 3 has a sensitivity of 86.0% and a specificity of 83.0% for predicting generalized anxiety disorder [53]. Scores ≥ 5 on the PHQ-2 and GAD-2 were considered “red flags” (indicating a very high probability of a depressive or anxiety disorder, respectively, as these scores were higher than 99% of those of the normative general population) [47,51].

The overall PHQ-4 score serves as a general marker of psychological distress, indicating impairment and disability as well as symptom burden. A score of 0–2 is considered normal, 3–5 is interpreted as mild symptoms, 6–8 is considered moderate, and 9–12 is associated with severe symptoms [51]. Löwe et al. recommend a “yellow flag” for a PHQ-4 score ≥ 6, as these scores are higher than 95% of those of the normative general population, and a “red flag” for a PHQ-4 score ≥ 9, indicating the presence of psychological distress, as these scores are higher than 99% of those of the general normative population [47,51].

### 2.5. Brief Resilience Scale (BRS)

The BRS was used to assess how resilience affected the mental health outcomes of the participants. Smith et al. [54] was the first to describe the BRS, which was developed to assess the ability to recover from stress. The reliability of the BRS in the German population, which was analyzed by Chmitorz et al., was α = 0.85, which is in line with the results of the original validation study by Smith et al. [55,56]. The BRS includes six items that are rated on a 5-point Likert scale: 1 = strongly disagree, 2 = disagree, 3 = neutral, 4 = agree, and 5 = strongly agree. The first, third, and fifth items are positively worded, and the second, forth, and sixth items are negatively phrased. The total score is the average of all six items [56].

### 2.6. Statistical Analyses

The demographic characteristics of the study population were analyzed using descriptive statistics (frequencies, means, and standard deviations). Differences between study respondents and nonrespondents were examined using the χ^2^ test and the Mann–Whitney U-test.

Univariate analysis was used to identify variables with proper discriminatory value to detect high (PDI ≥ 32) or very high levels of disability (PDI ≥ 43). The following dependent variables were evaluated: high pain-induced disability, which was defined as disability scores equal to or higher than the median of the study population, i.e., PDI ≥ 32 (controls (co): PDI < 32), and very high pain-induced disability, which was defined as a disability score equal to or greater than the 75th percentile of the study population, i.e., PDI ≥ 43 (co: PDI < 43). Age (continuous variable (cv)), having a stable partnership (co: not having a stable partnership), living alone (co: not living alone), and a tertiary level of education (co: up to a secondary level of education) were used as demographic predictors of pain-induced disability. The following pandemic-specific variables were analyzed: large reduction in social network (co: no reduction or mild reduction), period of social distancing ≥ 15 days (co: social distancing period < 15 days), and isolation or quarantine (co: no isolation or quarantine). A cut-off value of 15 days for the social distancing period was chosen in accordance with published data on the duration for imposed quarantine [57]. The following disease-specific variables were analyzed to examine their predictive power: time since the endometriosis diagnosis (in years, continuous variable), age at diagnosis (in years, continuous variable), time since the onset of pain (in years, continuous variable), delay in the diagnoses (in years, continuous variable), continuous pain (co: patients with pain peaks), and the number of pain localizations (continuous variable, six localizations were assessed). Pain intensity prior to isolation or quarantine and current pain levels were included: dysmenorrhea, noncyclic pain, dyspareunia, dyschezia, dysuria, lower back pain, and general pain (the mean pain intensity with regard to all previously named pain localizations). This parameter was measured using either continuous (VAS_P_ or VAS_C_) or ordinal variables for no or low level of pain (VAS_PL_, VAS_CL_), a moderate level of pain (VAS_PM_, VAS_CM_; co: VAS_PL_, VAS_CL_), and a severe level of pain (VAS_PS_, VAS_CS_; co: VAS_PL_, VAS_CL_).

Variables with *p*-values less than 0.25 in the univariate regression model were analyzed using backward stepwise regression to select predictors for inclusion in the final multivariate logistic regression model to assess the independence of the predictors of pain-induced disability [58,59]. Data were expressed as odds ratio (OR), variance (Nagelkerke R^2^), *p*-value, and 95% confidence intervals (95% CIs).

All of the tests were two-tailed, with a significance level of *p* < 0.05. All analyses were conducted using SPSS^®^ software Version 24 (IBM SPSS Statistics for Windows, Version 24.0. Armonk, NY, USA).

## 3. Results

### 3.1. Demographic Characteristics of the Study Group

A total of 413 participants met the inclusion criteria and accessed the survey, but only 277 (67.7%) of them answered the questions on the PDI. To determine whether there were any differences between the participants who did (respondents) and did not (nonrespondents) answer the PDI questions, we analyzed the data collected from both groups on their demographic and clinical characteristics, as well as their mental health (PHQ-4 and BRS scores) (Table S1). We detected no significant differences between the responders and nonresponders, except for the item “current self-care” of the PDI questionnaire (Table S1).

### 3.2. Level of Self-Reported Pain-Induced Disability in Women with Endometriosis during the COVID-19 Pandemic in Comparison with Previously Described Populations

The mean level of pain-induced disability was 31.61 (SD = 15.82) in this study population of 277 women with endometriosis. The level of disability was significantly higher (*p* < 0.001) than the level reported in a normative female German population at 6.9 (SD = 11.1; N = 1368) [60], and it was significantly lower than the level of pain-induced disability of patients awaiting hip surgery, but it was not significantly lower compared to the disability of other populations with chronic pain [61,62,63,64] (Table 1).

### 3.3. Identification of Predictors of Pain-Induced Disability

The ability of the selected independent variables to predict the odds of having high pain-induced disability (a PDI score ≥ 32 represented the level equal to or higher than the median level of the study group) or very high pain-induced disability (a PDI score ≥ 43 represented the level equal to or higher than the level of the highest quartile of the study group) were assessed using univariate logistic regression analyses.

**Figure 1:**
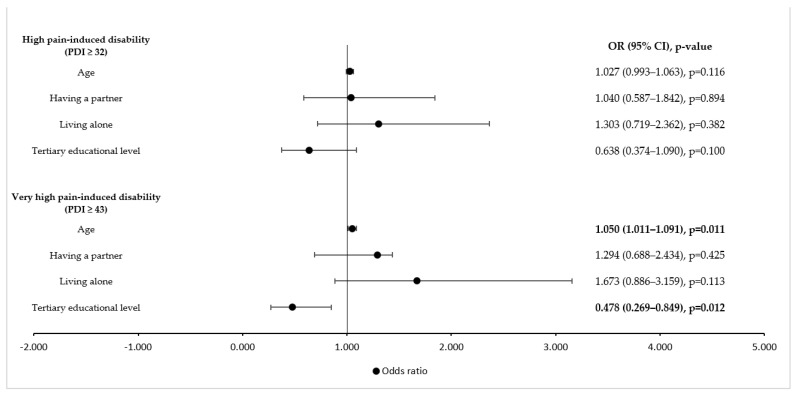
Influence of demographic factors on high and very high pain-induced disability (univariate logistic regression analysis). PDI = Pain Disability Index; OR = odds ratio; CI = confidence interval. Values in bold indicate statistical significance, as the level of statistical significance was set to *p* < 0.05.

Figure 1 shows the influence of the demographic variables on the participants’ resilience scores. A high educational level reduced the odds of very high pain-induced disability by 52.2%, while an older age increased the odds. The other demographic variables that were assessed had no impact on high or very high pain-induced disability.

**Figure 2:**
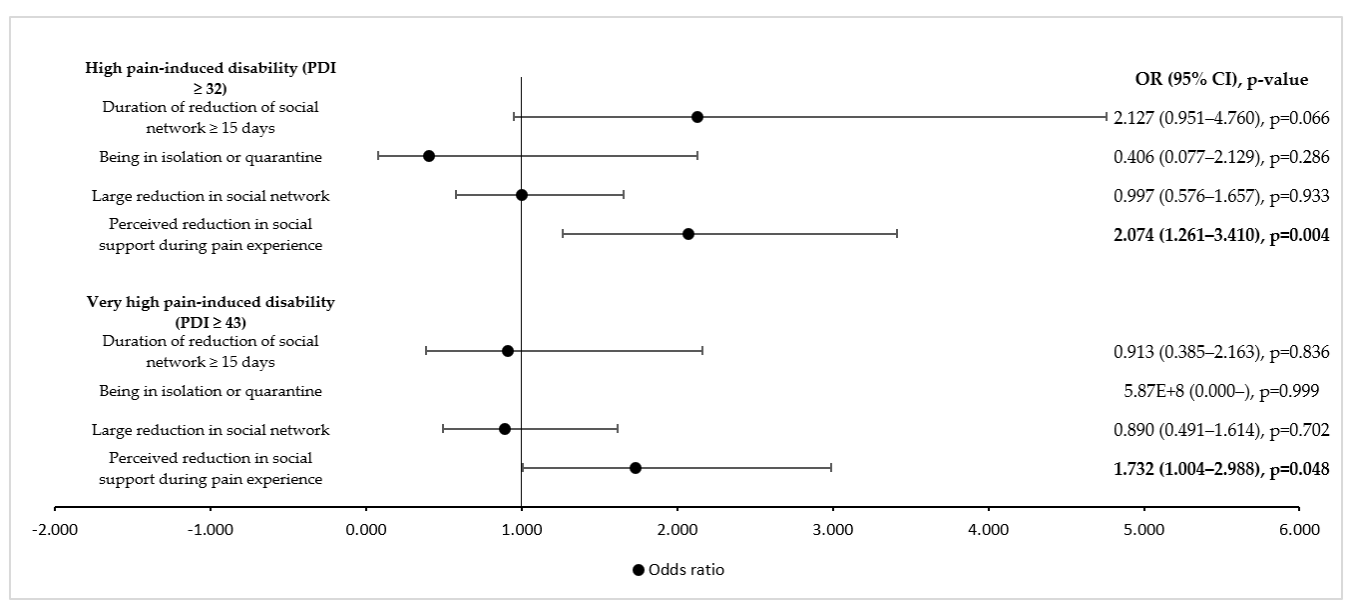
Influence of pandemic-specific factors on high and very high pain-induced disability (univariate logistic regression analysis). PDI = Pain Disability Index; OR = odds ratio; CI = confidence interval. Values in bold indicate statistical significance, as the level of statistical significance was set to *p* < 0.05.

Pandemic-specific factors, such as the duration of a reduced social network, being in isolation or quarantine, and the level of reduction of social contacts did not significantly influence the pain-induced disability of the participants in our study (Figure 2). Perceived reduction of social support during the experience of pain led to a significant increase in high and very high pain-induced disability (Figure 2). 

**Figure 3:**
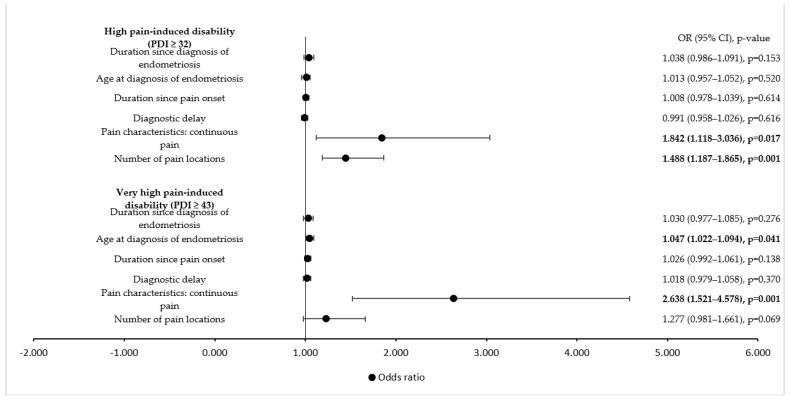
Influence of endometriosis-specific history on high and very high pain-induced disability (univariate logistic regression analysis). PDI = Pain Disability Index; OR = odds ratio; CI = confidence interval. Values in bold indicate statistical significance, as the level of statistical significance was set to *p* < 0.05.

An endometriosis-related medical history showed no influence on disability with respect to factors, such as the time since the diagnosis of endometriosis or delay in the diagnosis, whereas continuous pain significantly increased the odds of high and very high pain-induced disability. The higher the number of pain localizations, the higher the odds were for high pain-induced disability (Figure 3). 

Pain intensity prior to and during the pandemic were entered into the univariate logistic regression as continuous and ordinal variables (current pain) when moderate or severe levels of pain were compared to no pain or low levels of pain (Table 2). Current pain was more strongly associated with current pain-induced disability than pain before the pandemic was. Moderate dysmenorrhea and lower back pain did not significantly increase the odds of high or very high pain-induced disability, whereas other symptoms of pain (dyspareunia, dysuria, dyschezia, noncyclic pain, and global pain level) increased the odds of high or very high pain-induced disability even at a moderate level of pain (VAS 45–74 mm) (Table 2). 

**Figure 4:**
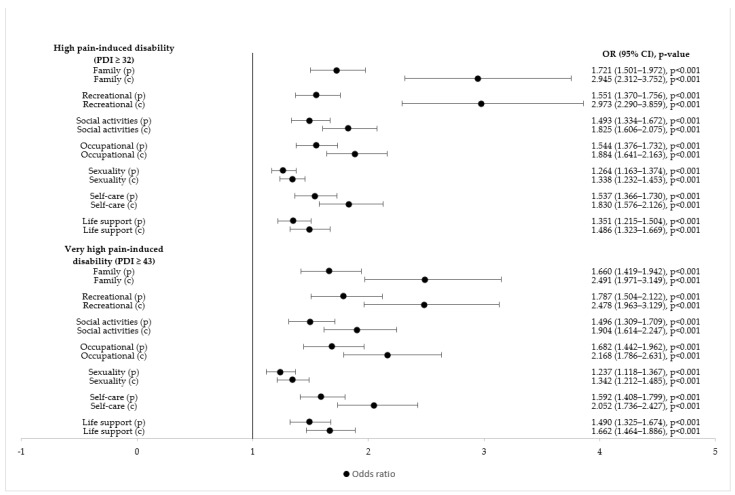
Forest plot (univariate logistic regression analysis) of the influence of single items of daily activities (prior to the pandemic and current activities) on high and very high pain-induced disability (univariate logistic regression analysis). PDI = Pain Disability Index; OR = odds ratio; CI = confidence interval, i/q = isolation/quarantine; p = prior to i/q; c = current.

The current single items pertaining to pain-induced disability related to activities of daily living showed higher odds for high or very high pain-induced disability than did the experience of impairment in activities of daily living prior to the pandemic. Current impairment in family activities and impaired participation in recreational activities were the items associated with the highest increase in the odds of experiencing high or very high pain-induced disability (Figure 4).

Adverse mental health outcomes, specifically depression to a higher extent than anxiety, increased the odds for high or very high pain-induced disability. The likelihood of experiencing a high level of pain-induced disability were 6.453-fold higher among women with a high risk for depression (PHQ-2 ≥ 5) and 2.685-fold higher in women with a high risk for generalized anxiety disorder (GAD-2 ≥ 5) (Table 3). Resilience decreased the odds for high pain-induced disability by 38.9% and for very high pain-induced disability by 34.3% (Table 3). 

To evaluate the independence of the above-mentioned predictor variables, we performed multivariate logistic regression analysis and included the strongest predictors of resilience in the univariate analyses (*p* < 0.25). In order to avoid model overfitting and to minimize redundancy of the information, the single subscores from the PDI were excluded from the analyses (as they were part of the global score), as well as the pain prior to the pandemic and the current global pain (asthe global pain was computed using the current pain characteristics).

For a high level of pain-induced disability (a PDI score ≥ 32 represented those with PDI levels equal to or higher than the median of the study population), the multivariate logistic regression model included age, duration of a reduced social network, educational level, perceived social support for pain, length of time before the diagnosis of endometriosis, continuous pain, number of pain localizations, current dysmenorrhea (cv), current noncyclic pain (cv), current dyspareunia (cv), current dyschezia, current dysuria (cv), current lower back pain (cv), resilience (cv), and PHQ-4 score (cv). Older age (OR 1.063, 95% CI 1.010–1.120, *p* = 0.020), dysmenorrhea (OR 1.015, 95% CI 1.005–1.026, *p* = 0.005), dysuria (OR 1.014; 95% CI 1.001–1.027, *p* = 0.029), lower back pain (OR 1.018, 95% CI 1.007–1.029, *p* = 0.001), and impaired mental health (OR 1.271, 95% CI 1.134–1.425, *p* < 0.001) were risk factors for high pain-induced disability. The final multivariate logistic regression model (*n* = 208) explained 42.7% of the variance and demonstrated a 75% sensitivity for predicting a PDI score ≥ 32 in women with endometriosis. 

Age, living alone, educational level, perceived social support for pain, age at the time of the endometriosis diagnosis, time since the onset of pain, continuous pain, number of pain localizations, current dysmenorrhea (cv), current noncyclic pain (cv), current dyspareunia (cv), current dyschezia (cv), current dysuria (cv), current lower back pain (cv), resilience (cv), and PHQ-4 score (cv) were identified as possible predictors of very high pain-induced disability (scoring ≥43 or at least at the 75th percentile of the study population on the PDI scale). A total of 207 participants were included in the multivariate logistic regression model. Older age (OR 1.080; 95% CI 1.025–1.139; *p* = 0.004), dysmenorrhea (OR 1.021; 95% CI 1.008–1.034; *p* = 0.002), dyschezia (OR 1.016; 95% CI 1.004–1.028; *p* = 0.009), and impaired mental health (OR 1.131; 95% CI 1.007–1.269; *p* = 0.037) significantly increased the odds of experiencing very high pain-induced disability. The final multivariate logistic regression model explained 34.2% of the variance and demonstrated a 78.7% sensitivity for predicting a high level of disability (PDI ≥ 43) in women with endometriosis. 

## 4. Discussion

We showed previously that the global pain-induced disability significantly decreased during the first wave of the pandemic compared to pre-pandemic levels, as up to 48% of women with endometriosis experienced decreased disability [13]. Nonetheless, more than 40% of the participants experienced increased disability during the pandemic [13]. In order to be able to identify women at risk for increased disability, it is crucial to characterize potential protective factors as well as possible threats to the physical well-being and to social functioning. To our knowledge, this is the first study to assess the complex associations of demographic, disease-specific, COVID-19-pandemic-specific, and psychological factors, as well as resilience with pain-induced disability in women with endometriosis. We used the PDI, a self-report instrument, to assess the level of impairment in various aspects of daily life. 

Women with endometriosis experienced significant interference of pain with their daily life activities, compared with that in a normative German population [60]. In contrast, the global disability level did not differ from that of other populations of German patients with chronic pain [62,63,64]. Only a group of patients who were awaiting hip surgery scored significantly higher than the population of the present study [61]; nevertheless, all the participants in our sample had already received surgery (as a requirement to enter the study).

Several demographic factors, such as age, living alone, and educational level were identified as potential predictors of pain-induced disability in our study population. However, when age was adjusted for other factors, it was the only significant independent demographic predictor of disability in women with endometriosis. Our results corroborate previous findings, as advanced age was a risk factor for disability, physical functioning, and social relationships in other persons with chronic pain [29,65]. As shown in this study, the association of pain intensity with perceived disability and control over one’s life in women with endometriosis seemed to be significantly stronger with increasing age. In contrast to other studies [65,66], the present study did not identify educational level as an independent predictor of pain-induced disability in women with endometriosis. 

Pandemic-specific factors, such as the duration or level of reduction of a social network and perceived social support for pain were not identified as independent predictors of pain-induced disability in women with endometriosis during the first wave of the COVID-19 pandemic in Germany. Social support is an essential resource for helping people, especially women, overcome hardships, such as natural disasters [67,68]. Therefore, it is not astonishing that functional outcomes were better in persons experiencing chronic pain who had good social support systems than in their counterparts with limited social interactions [69,70,71,72]. Social isolation can be a significant predictor of pain-induced disability in persons with lower back pain, presumably by negatively influencing self-efficacy and the way patients with chronic pain cope with the disease [73]. In contrast, perceived social support related to the experience of pain showed no significant influence on pain-induced disability when it was adjusted for multiple potential predictors. Social support and resilience were acknowledged as protectors of mental health during the COVID-19 pandemic [19,74,75,76]. Taken together, these results suggest that social support may act as a mediator of pain-induced disability through resilience and mental health rather than an independent factor. 

The impact of pain on daily activity has been recognized as an indicator of well-being and quality of life in patients with chronic pain [77]. In this study, we had the unique opportunity to assess the influence of various aspects of daily life on the level (high or very high level) of perceived pain-induced global disability. Previous studies have often evaluated the impact of one or two distinctive aspects of life on pain-induced disability, such as work-related disability [16,78] or sexual dysfunction [9]. In contrast, by using the PDI, we compared the influence of pain on seven different facets of life [27,46]. A recent study published by Leuenberger and colleagues found that women with endometriosis were often severely impaired in multiple areas of daily life, with up to one third of them recognizing severe impairment in up to nine distinct areas of daily life [79]. Impaired role performance and social functioning has been described previously as well [80,81]. In this study, we assessed the influence of single items of the PDI on the level of global disability (equal to or higher than the median or the 75th percentile). We were able to identify specific factors with an additional negative impact on the remaining items, thereby increasing the odds of higher disability levels. Univariate logistic regression analyses revealed that greater family dysfunction and recreational aspects of life increased the odds of high (PDI score ≥ median) or very high (PDI score ≥ 75th percentile) perceived global pain-induced disability by at least two to three-fold. Thus, we suggest that the inability to properly engage in daily family and recreational activities are key triggers for additional functional and social disabilities and may lead to powerlessness, a resigned attitude, and the feeling of an interrupted life, as described previously [2,81,82]. These effects might have been amplified during the pandemic, when social restriction in order to contain the viral spread resulted in a concentration of activities around ones’ household. Evaluation of disability is a complex process. Clinicians often concentrate on loss of function of organs or physical abilities rather than on interference with family life or recreational activities. As reported in this study, healthcare professionals might miss the most influential aspects causing disability in women with endometriosis. Thus, being able to develop and maintain family bonds by spending and enjoying quality time during family activities and recreational activities seems to be the most important source of strength and empowerment of supportive forces for the individual to prevent adverse outcomes caused by chronic pain. 

Impaired participation in social activities, such as gatherings with friends or acquaintances, and occupational activities influenced global pain-induced disability to a lower extent, signaling than these activities are not as important as the ability for women with endometriosis to participate in family and recreational activities. Nevertheless, impaired socialization is a well-recognized factor with a negative influence on the quality of life of these patients [79,80,81]. Due to chronic pain, women with endometriosis report impaired occupational functioning, as they often cannot reach full professional fulfillment [79,80,81]. The ability to perform in the work setting is a major social concern. Occupational pain-induced disability may lead to absenteeism, reduced work productivity and effectiveness, and impaired professional growth, which increase with disease severity and perceived disability [15,16,20,78]. Surprisingly, impaired sexual function had the lowest effect on global pain-induced disability. It is possible that women who repeatedly experience dyspareunia avoid engaging in sexual intercourse. 

Clinicians should be aware that the avoidance of daily activities due to pain may lead to a vicious cycle of pain, pain-related fear, and pain-induced disability, which may pave the way for the chronification of pain and maintenance of chronic pain disability through avoidance behavior, as observed in patients with chronic musculoskeletal pain conditions [33]. The most important information for society and women with endometriosis is that avoidance behavior in response to pain or pain-induced disability may be resistant to extinction [33,83].

Pain-related factors were identified as predictors for perceived disability. A higher number of pain localizations (more than five localizations: OR 12.4, 95% CI 5.4–28.2, *p* < 0.001) resulted in higher work-related disability in Finnish women with chronic musculoskeletal pain [66], and in a lower quality of life in affected women in the US [84]. We confirmed these results using univariate analysis, as the study respondents showed significantly higher disability levels (equal to or higher than the median) with an increasing number of symptoms, but this effect disappeared when it was adjusted for other influential factors. We also observed a positive relationship between previous and current pain intensity and pain-induced disability in all of the assessed pain symptoms (dysmenorrhea, dyspareunia, dyschezia, dysuria, noncyclic pain, and lower back pain), whereas the current pain intensity was, as expected, more strongly correlated with current disability than with the previous pain. 

The relationship between type of pain and pain intensity with pain-induced disability is of particular importance for clinicians’ estimates of the importance and urgency of therapeutic procedures. Healthcare professionals must be aware of the types of pain that may trigger a decline in physical and social well-being. Clinicians must consider differences between physicians’ and patients’ perceptions of pain intensity and the resulting impairment in the various aspects of their daily lives. One should be careful when comparing pain levels to other known chronic pain conditions, as endometriosis is an individual disease, requiring tailored treatment. In this study, we found that women experiencing even moderate levels of dyschezia, dysuria, or noncyclic pain seemed to be especially susceptible to pain-induced disability, while only severe dysmenorrhea had a significant influence on high or very high pain-induced disability, compared to women experiencing no pain or low pain levels. We assume that moderate pain affecting daily physiological processes, such as urination and defecation, may increase the sense of hopelessness and fear of losing control over one’s own body, generating feelings of greater vulnerability among affected women compared to their unaffected counterparts. There is an increasing body of evidence for the importance of being able to manage and control pain with respect to coping with pain and perceived self-efficacy [25]. Moderate dysmenorrhea seems to be more manageable than moderate dysuria or dyschezia. This might also be related to the possibility of controlling the onset of menstruation using medication, such as contraceptives, whereas urination and defecation are physiological processes that can be voluntarily controlled for only a limited period. Thus, the functional outcomes of the participants who experienced moderate monthly pain were better than the outcomes of those who experienced daily pain. Chronic pain, which appears to be uncontrollable by persons who experience negative sensations, may enhance feelings of helplessness and adverse mental outcomes [85].

We found that dysmenorrhea was an independent predictor of high and very high pain-induced disability (women with disability equal to or higher than the median and equal to or higher than the 75th percentile). Dyschezia, dysuria, and lower back pain proved to be moderators of pain-induced disability, as they significantly increased the odds of having high (PDI ≥ 32) or very high disability (PDI ≥ 43) in our study group. A recent study revealed that dyschezia and back pain in women with endometriosis are significantly associated with a higher number of other pain contributors related to central nervous system sensitization, such as migraines, fibromyalgia, chronic fatigue syndrome, depression, and anxiety [86]. 

Central sensitization is characterized by an increased excitability of central nociceptive circuits, caused by hypersensitivity to sensory inputs, even in the absence of inflammation or other peripheral noxious stimuli [87]. High scores on the central sensitization scale were positively correlated with high disability in a Dutch study of patients with chronic spinal pain [22], other musculoskeletal pain disorders [21,23], or pain-related disability after oncological surgery [24]. Taken together, women with endometriosis who experience high disability may be at risk for central sensitization and at a higher risk for chronification of their pain; therefore, they may benefit from additional therapeutic options with centrally acting treatments, such as pregabalin or duloxetine [87]. We also suggest that women with endometriosis and high disability should be screened for co-existing conditions with chronic pain, such as fibromyalgia or chronic fatigue, and should be managed by a multidisciplinary team of healthcare professionals. 

Resilience is described as a trait, a process, or an outcome, depending on the definition [88,89]. In times of hardship, resilience enables people to recover from adversity or stressful events [54,55,88,90,91,92]. In the context of chronic pain, studies have described resilience as a personal resource for managing pain effectively [69,92]. Resilience positively influences the perceived physical disability of patients with chronic pain and pain-related long-term morbidity and mortality [30,90,92]. Given that endometriosis is a chronic pain condition, resilience in stressful situations, such as chronic pain, could improve long-term outcomes with respect to physical and social functioning. Resilience in response to pain has been acknowledged as a mediator of the relationship between pain and physical functioning in persons with chronic pain [93]. People with chronic pain who demonstrate resilience as part of their coping strategies show a better adjustment to pain and a higher level of daily functioning compared to those who experience pain as a threat rather than a challenge [77]. Thus, we examined the possible effect of resilience on pain-related functional disability. When adjusted for other factors, such as mental health, we did not find a direct significant association between resilience and pain-induced disability. Nevertheless, as reported previously, resilience is a protective factor against adverse psychological outcomes [74,75]. Resilience has been described as a predictor of coping with threats, and a predictor of mental well-being in people experiencing natural disasters and health crises [42,43,94,95,96]. Thus, resilience seems to be a mediator of pain-induced disability, by positively influencing depression and anxiety in patients with chronic pain. 

Psychological distress was an independent, and the most influential predictor of high levels of pain-induced disability in this study of German women with endometriosis (an OR 1.271 for those scoring higher than the median and an OR 1.131 for those with disability levels equal to or higher than the 75th percentile of the study population). Univariate analyses showed that depressive symptoms had a greater impact on daily high and very high pain-induced disability than anxiety did. The experience of pain and the ability to manage it is shaped by the way a person interprets the situation [25], and depressive symptoms seem to play a major role in modifying the experiences and subsequent social and functional consequences of women with endometriosis. 

Pain-induced disability is an independent risk factor for adverse mental health outcomes in women with endometriosis [19]. Thus, we suggest that in women with endometriosis, physical and psychological adverse outcomes appear to have a reciprocal interaction, forming a vicious cycle of pain maintenance and reinforcement. Our results corroborate previous observations, such as those first proposed by Sharp and Havery for other chronic pain conditions, such as fibromyalgia [31,97], which has a deleterious impact on the patient’s quality of life. Anxiety and depression seem to interfere with one’s ability to cope effectively with pain. Pain and depression share common neurological pathways, leading to helplessness and fear of physical activity [85]. The detrimental impact of adverse mental health outcomes and pain-induced disability has been described in other chronic pain conditions [25], such as chronic neck pain [98] and chronic shoulder pain [99,100]. Psychological distress has been identified as a mediator of the relationship between pain and pain-induced disability in persons with back pain [101,102], which is also a symptom of endometriosis. Taken together, effective treatment of negative outcomes of pain, such as impaired physical and social functioning, should be managed by a multidisciplinary team and a multimodal therapy plan, in order to address mental health along with other treatment approaches, such as physical therapy, integrative medical approaches, or surgical or pharmacological treatment [103].

### Limitations

We wish to consider the current findings within the context of certain limitations. As we recruited the study participants online, this approach might have generated a self-selection effect, as women experiencing a greater disease burden may sign in Facebook support groups and may have had access to the questionnaire. Nonetheless, a recent systematic review found that Facebook-recruited samples are similarly representative as samples recruited via traditional methods [104]. Nevertheless, social desirability bias may have been a study limitation, as the patients responded directly to the questionnaire.

Second, we observed a significant drop in the sample size of the participants, as 413 accessed the questionnaire, but only the 277 who answered the questionnaire regarding pain-induced disability were analyzed. Nevertheless, we did not observe any statistically significant difference between the “Respondents” and “Non-respondents to the PDI”, except for the item “self-care” of the PDI questionnaire. Thus, the drop in sample size did not influence the results of the study. 

Third, we did not assess the specific medication of the study participants, such as the intake of progestins. Moreover, we did not assess the period of time between surgery related to endometriosis and the participation to the survey. These factors could have influenced the results as well.

Next, recall bias may have influenced the results of the study. While recall is a common tool in the field of endometriosis, and previous findings proved that women with endometriosis were relatively accurate in their recall of pain [105], there are no published data with respect to the accuracy of recall regarding pain-induced disability. 

Finally, this study’s results are based on a cross-sectional design; thus, we were not able to analyze the potential relationship between pain-induced disability levels in response to the development of the COVID-19 pandemic. Nevertheless, as pandemic-specific factors did not independently influence pain-induced disability, the design of the study did not impact the results.

## 5. Conclusions

It is crucial to identify women at risk for pain-induced disability in order to reduce long-lasting adverse outcomes in women with endometriosis. Interestingly, we did not find that pandemic-specific factors significantly and independently influenced pain-induced disability during the first wave of the COVID-19 pandemic in Germany. Nevertheless, the increased odds for increased pain-induced disability as a consequence of impairment in family and recreational daily activities during the first wave of the pandemic might have been an effect of government-imposed social distancing measures. We found that several endometriosis-associated pain conditions, such as dysmenorrhea, dysuria, dyschezia, and lower back pain were independent risk factors for impaired physical and social functioning. We also observed a positive association between high levels of mental distress and the perception of high levels of pain-induced disability. In order to understand the dynamics and impact of persistent pain on daily life and initiate proper psychological interventions for preventing long-lasting pain-related physical and psychological sequalae, we recommend continuous assessment of pain-induced disability and mental health in women with endometriosis throughout treatment and follow-up.

## Figures and Tables

**Table 1:** Pain-induced disability in different German populations.

	M (PDI Level)	SD	*n*	*p*-Value
Current study	31.61	15.82	277	n.a.
Mewes et al. [60]	6.9	11.1	1368	**<0.001**
Nilges et al. [61]	37.03	13.73	42	**0.036**
Luka-Krausgrill et al. [62]	31.5	15.39	154	0.944
Saile and Dieterich [63]	30.5	13.9	40	0.674
Saile and Schmitz [64]	32.58	15.13	82	0.622

PDI = Pain Disability Index; M = mean; SD = standard deviation; *n* = number of participants; n.a. = not applicable. Values in bold indicate statistical significance, as the level of statistical significance was set to *p* < 0.05.

**Table 2:** Influence of pain intensity on high and very high pain-induced disability (univariate logistic regression analysis).

	High Pain-Induced Disability (PDI ≥ 32)		Very High Pain-Induced Disability (PDI ≥ 43)	
	*p*-Value	OR (95% CI)	*p*-Value	OR (95% CI)
Dysmenorrhea prior to i/q				
VAS_P_	**0.001**	**1.015** **(1.006–1.024)**	**0.013**	**1.013** **(1.003–1.023)**
VAS_PM_ (co: VAS_PL_)	0.589	1.221 (0.591–2.2524)	0.982	1.011 (0.402–2.543)
VAS_CS_ (co: VAS_PL_)	**<0.001**	**3.664** **(1.899–7.071)**	**0.010**	**2.769** **(1.277–6.004)**
Noncyclic pain prior to i/q				
VAS_P_	**<0.001**	**1.021** **(1.011–1.031)**	**<0.001**	**1.023** **(1.012–1.035)**
VAS_PM_ (co: VAS_PL_)	**<0.001**	**3.026** **(1.713–5.344)**	**0.002**	**2.876** **(1.455–5.685)**
VAS_CS_ (co: VAS_PL_)	**0.001**	**2.890** **(1.505–5.548)**	**0.002**	**3.281** **(1.548–6.956)**
Dyspareunia prior to i/q				
VAS_P_	**0.005**	**1.011** **(1.003–1.019)**	**0.008**	**1.012** **(1.003–1.021)**
VAS_PM_ (co: VAS_PL_)	**0.028**	**1.910** **(1.074–3.397)**	1.182	1.578 (0.807–3.086)
VAS_CS_ (co: VAS_PL_)	**0.021**	**2.174** **(1.121–4.213)**	**0.016**	**2.423** **(1.181–4.972)**
Dysuria prior to i/q				
VAS_P_	**<0.001**	**1.021** **(1.011–1.031)**	**0.002**	**1.015** **(1.006–1.025)**
VAS_PM_ (co: VAS_PL_)	**0.005**	**2.824** **(1.378–5.788)**	**0.041**	**2.132** **(1.030–4.413)**
VAS_CS_ (co: VAS_PL_)	**0.005**	**4.008** **(1.529–10.512)**	**0.013**	**3.015** **(1.265–7.188)**
Dyschezia prior to i/q				
VAS_P_	**<0.001**	**1.015** **(1.007–1.024)**	**<0.001**	**1.018** **(1.009–1.028)**
VAS_PM_ (co: VAS_PL_)	0.055	1.824 (0.988–3.370)	**0.001**	**3.018** **(1.543–5.903)**
VAS_CS_ (co: VAS_PL_)	**0.005**	**2.656** **(1.348–5.233)**	**0.007**	**2.667** **(1.305–5.449)**
Lower back pain prior to i/q				
VAS_P_	**<0.001**	**1.021** **(1.012–1.029)**	**<0.001**	**1.020** **(1.011–1.030)**
VAS_PM_ (co: VAS_PL_)	0.650	1.159 (0.613–2.193)	0.405	1.417 (0.625–3.213)
VAS_CS_ (co: VAS_PL_)	**<0.001**	**3.895** **(2.176–6.971)**	**<0.001**	**3.930** **(2.006–7.701)**
Global pain experience prior to i/q				
VAS_P_	**<0.001**	**1.048** **(1.030–1.066)**	**<0.001**	**1.045** **(1.025–1.066)**
VAS_PM_ (co: VAS_PL_)	**<0.001**	**4.347** **(2.370–7.973)**	**0.003**	**3.218** **(1.498–6.912)**
VAS_CS_ (co: VAS_PL_)	**0.001**	**7.845** **(2.376–25.897)**	**<0.001**	**11.857** **(3.802–36.976)**
Current dysmenorrhea				
VAS_C_	**<0.001**	**1.021** **(1.013–1.030)**	**<0.001**	**1.029** **(1.017–1.041)**
VAS_CM_ (co: VAS_CL_)	0.327	1.421 (0.704–2.872)	0.402	1.566 (0.548–4.476)
VAS_CS_ (co: VAS_CL_)	**<0.001**	**5.577** **(2.951–10.539)**	**<0.001**	**7.510** **(3.176–17.757)**
Current noncyclic pain				
VAS_C_	**<0.001**	**1.027** **(1.018–1.037)**	**<0.001**	**1.032** **(1.020–1.044)**
VAS_CM_ (co: VAS_CL_)	**<0.001**	**2.977** **(1.638–5.410)**	**0.037**	**2.380** **(1.056–5.366)**
VAS_CS_ (co: VAS_CL_)	**<0.001**	**7.031** **(3.620–13.658)**	**<0.001**	**9.199** **(4.263–19.850)**
Current dyspareunia				
VAS_C_	**<0.001**	**1.017** **(1.009–1.024)**	**<0.001**	**1.016** **(1.007–1.024)**
VAS_CM_ (co: VAS_CL_)	**0.003**	**2.468** **(1.348–4.516)**	0.248	1.538 (0.741–3.194)
VAS_CS_ (co: VAS_CL_)	**<0.001**	**3.250** **(1.703–6.201)**	**<0.001**	**4.091** **(2.049–8.168)**
Current dysuria				
VAS_C_	**<0.001**	**1.023** **(1.014–1.033)**	**<0.001**	**1.018** **(1.009–1.027)**
VAS_CM_ (co: VAS_CL_)	**0.002**	**3.033** **(1.507–6.105)**	**0.009**	**2.576** **(1.264–5.250)**
VAS_CS_	**<0.001**	**4.892** **(2.008–11.923)**	**0.003**	**3.300** **(1.490–7.305)**
Current dyschezia				
VAS_C_	**<0.001**	**1.025** **(1.016–1.034)**	**<0.001**	**1.027** **(1.017–1.037)**
VAS_CM_ (co: VAS_CL_)	**<0.001**	**2.911** **(1.597–5.306)**	**<0.001**	**3.474** **(1.736–6.952)**
VAS_CS_ (co: VAS_CL_)	**<0.001**	**6.262** **(2.876–13.634)**	**<0.001**	**6.645** **(3.142–14.056)**
Current lower back pain				
VAS_C_	**<0.001**	**1.023** **(1.015–1.031)**	**<0.001**	**1.023** **(1.013–1.033)**
VAS_CM_ (co: VAS_CL_)	0.718	1.135(0.571–2.257)	0.652	1.240 (0.487–3.156)
VAS_CS_ (co: VAS_CL_)	**<0.001**	**5.862** **(3.208–10.713)**	**<0.001**	**4.909** **(2.411–9.997)**
Current global pain experience				
VAS_C_	**<0.001**	**1.057** **(1.040–1.075)**	**<0.001**	**1.056** **(1.036–1.077)**
VAS_CM_ (co: VAS_CL_)	**<0.001**	**4.562** **(2.507–8.303)**	**0.003**	**3.233** **(1.495–6.990)**
VAS_CS_ (co: VAS_CL_)	**<0.001**	**51.806** **(6.685–401.457)**	**<0.001**	**30.436** **(9.433–98.206)**

PDI = Pain Disability Index; OR = odds ratio; CI = confidence interval; i/q = isolation or quarantine; VAS_P_ = previous pain level, continuous variable; VAS_PL_ = previous low pain level (VAS = 0–44); VAS_PM_ = previous moderate pain level (VAS = 45–74); VAS_PS_ = previous severe pain level (VAS = 75–100); VAS_C_ = current pain level, continuous variable; VAS_CL_ = current “no or low pain” level (VAS = 0–44); VAS_CM_ = current “moderate pain” level (VAS = 45–74); VAS_CS_ = current “severe pain” level (VAS = 75–100); co = controls. Values in bold indicate statistical significance, as the level of statistical significance was set to *p* < 0.05.

**Table 3:** Influence of mental health on high and very high pain-induced disability (univariate logistic regression analysis).

	High Pain-Induced Disability (PDI ≥ 32)		Very High Pain-Induced Disability (PDI ≥ 43)	
	*p*-Value	OR (95% CI)	*p*-Value	OR (95% CI)
PHQ-2 cv	**<0.001**	**1.754** **(1.469–2.094)**	**<0.001**	**1.525** **(1.286–1.810)**
PHQ-2 ≥ 3	**<0.001**	**5.896** **(3.476–10.002)**	**<0.001**	**4.229** **(2.343–7.633)**
PHQ-2 ≥ 5	**<0.001**	**6.453** **(3.098–13.433)**	**0.002**	**2.643** **(1.433–4.878)**
GAD-2 cv	**<0.001**	**1.413** **(1.223–1.633)**	**<0.001**	**1.319** **(1.134–1.535)**
GAD-2 ≥ 3	**<0.001**	**2.780** **(1.696–4.558)**	**0.003**	**2.331** **(1.335–4.071)**
GAD-2 ≥ 5	**0.001**	**2.685** **(1.482–4.866)**	**0.006**	**2.290** **(1.263–4.152)**
PHQ-4 cv	**<0.001**	**1.310** **(1.198–1.433)**	**<0.001**	**1.232** **(1.127–1.348)**
PHQ-4 ≥ 6	**<0.001**	**4.536** **(2.702–7.615)**	**<0.001**	**3.125** **(1.777–5.495)**
PHQ-4 ≥ 9	**<0.001**	**5.813** **(2.925–11.554)**	**<0.001**	**3.550** **(1.949–6.465)**
BRS cv	**0.002**	**0.611** **(0.449–0.833)**	**0.018**	**0.657** **(0.464–0.930)**

PDI = Pain Disability Index; BRS = Brief Resilience Scale; GAD-2 = Generalized Anxiety Disorder Scale; PHQ-2 = Patient Health Questionnaire for Depression; PHQ-4 = Patient Health Questionnaire for Depression and Anxiety; OR = odds ratio; CI = confidence interval; cv = continuous variable; Values in bold indicate statistical significance, as the level of statistical significance was set to *p* < 0.05.

## Data Availability

The datasets analyzed during the current study are available from the corresponding author on reasonable request.

## Supplementary Materials

The following supporting information can be downloaded at: https://www.mdpi.com/article/10.3390/ijerph19148277/s1, Table S1: Differences between participants who did not complete (group “Non-respondents”) versus those who completed the PDI questionnaire (group “Respondents”).
