# Supplementary material for: Determinants of Pain-Induced Disability in German Women with Endometriosis during the COVID-19 Pandemic
Source: Int J Environ Res Public Health. 2022 Jul 6;19(14):8277. doi: 10.3390/ijerph19148277 (PMC9320034; doi:10.3390/ijerph19148277)
Supplement: Supplementary file 1 [file ijerph-19-08277-s001.zip › ijerph-1770061-supplementary.pdf]

**Supplementary Table S1. Differences between participants who did not complete (group "Non-respondents") versus those who completed the PDI questionnaire (group "Respondents").**

| Variables | Values | Non-respondents | Respondents | p-value |
|---|---|---|---|---|
| Demographic variables | | | | |
| Age | M (SD); N<br>Mdn (IQR) | 32.45 (6.92); 116<br>32.00 (27.00 – 36.00) | 31.94 (7.10); 277<br>31.00 (26.00 – 36.00) | 0.493[2] |
| Having a stable relationship | No in % (n/N)<br>Yes in % (n/N) | 70.6% (72/102)<br>29.4% (30/102) | 78.3% (217/277)<br>21.7% (60/277) | 0.116[1] |
| Living alone | No in % (n/N)<br>Yes in % (n/N) | 73.5% (86/117)<br>26.5% (31/117) | 80.1% (221/276)<br>19.9% (55/276) | 0.150[1] |
| Educational level | Up to secondary level in % (n/N)<br>Tertiary level in % (n/N) | 20% (1/5)<br>80% (4/5) | 29.5% (78/264)<br>70.5% (186/264) | 0.999[3] |
| Pandemic-specific variables | | | | |
| Duration of i/q | <15d in % (n/N)<br>≥15d in % (n/N) | 15.8% (16/101)<br>82.4% (85/101) | 10.5% (29/277)<br>89.5% (248/277) | 0.154[1] |
| Being in i/q | No in % (n/N)<br>Yes in % (n/N) | 6.0% (7/116)<br>94.0% (109/116) | 2.5% (7/277)<br>97.5% (270/277) | 0.087[1] |
| Reduction in social network | No to moderate reduction in % (n/N)<br>Large reduction in % (n/N) | 31.4% ((32/102)<br>68.6% (70/102) | 27.4% (76/277)<br>72.6% (201/277) | 0.452[1] |
| Perceived reduction in social support regarding pain experience during social isolation (by partner/ family/ friends) | No in % (n/N)<br>Yes in % (n/N) | 75.0% (3/4)<br>25.0% (1/4) | 61.2% (167/273)<br>38.8% (106/273) | 0.573[1] |
| Endometriosis-specific variables | | | | |
| Time since diagnosis (y) | M (SD); N<br>Mdn (IQR) | 3.94 (4.60); 95<br>2.00 (1.00 – 4.00) | 4.42 (4.84); 276<br>3.00 (1.00 – 5.00) | 0.289[2] |
| Age at diagnosis (y) | M (SD); N<br>Mdn (IQR) | 28.51 (6.77); 95<br>29.00 (24.00 – 33.00) | 27.53 (6.22); 276<br>27.00 (23.00 – 32.50) | 0.116[2] |
| Time since pain onset (y) | M (SD); N<br>Mdn (IQR) | 13.23 (7.63); 97<br>12.00 (7.00 – 18.00) | 14.02 (7.86); 277<br>13.00 (8.00 – 20.00) | 0.397[2] |
| Diagnostic delay (y) | M (SD); N<br>Mdn (IQR) | 9.39 (6.96); 95<br>9.00 (4.00 – 14.00) | 9.63 (6.88); 276<br>9.00 (4.50 – 14.00) | 0.920[2] |
| Pain characteristics | Pain peaks in % (n/N)<br>Continuous pain in % (n/N) | 66.7% (64/96)<br>33.3% (32/96) | 64.3% (178/277)<br>35.7% (99/277) | 0.670[1] |
| Number of pain localizations | M (SD); N<br>Mdn (IQR) | 5.00 (1.12), 39<br>5.00 (4.00 – 6.00) | 5.08 (1.16), 276<br>5.00 (5.00 – 6.00) | 0.580[2] |
| Pain intensity | | | | |
| Dysmenorrhoea prior to i/q | M (SD); N<br>Mdn (IQR) | 63.50 (33.98), 34<br>72.50 (49.00 – 96.00) | 65.25 (30.99), 250<br>73.50 (46.00 – 90.00) | 0.915[2] |
| Non-cyclical pain prior to i/q | M (SD); N<br>Median (IQR) | 49.61 (30.33), 38<br>46.50 (26.00 – 74.00) | 51.31 (26.83), 265<br>51.00 (31.00 – 72.00) | 0.681[2] |
| Dyspareunia prior to i/q | M (SD); N<br>Mdn (IQR) | 39.80 (32.16), 30<br>32.00 (11.00 – 59.00) | 45.00 (32.65), 254<br>45.50 (14.00 – 69.00) | 0.447[2] |

| | | | | |
|---|---|---|---|---|
| Dysuria prior i/q | M (SD); N<br>Mdn (IQR) | 30.30 (32.67), 33<br>11.00 (4.00 – 48.00) | 28.50 (28.92), 246<br>19.00 (3.00 – 48.00) | 0.914[2] |
| Dyschezia prior to i/q | M (SD); N<br>Mdn (IQR) | 46.14 (31.73), 35<br>48.00 (18.00 – 75.00) | 39.76 (30.94), 258<br>34.00 (12.00 – 66.00) | 0.257[2] |
| Lower back pain prior to i/q | M (SD); N<br>Mdn (IQR) | 57.14 (33.37), 37<br>63.00 (33.00 – 90.00) | 57.37 (32.30), 270<br>59.00 (33.00 – 87.00) | 0.896[2] |
| Global pain prior to i/q | M (SD); N<br>Mdn (IQR) | 46.09 (20.02), 26<br>46.92 30.17 – 59.83) | 47.29 (19.25), 211<br>47.50 (31.83 – 61.17) | 0.654[2] |
| Current dysmenorrhoea | M (SD); N<br>Mdn (IQR) | 28.67 (49.65), 3<br>0.00 (0.00 – 86.00) | 60.19 (33.57), 254<br>69.50 ( 30.00 – 87.00) | 0.160[2] |
| Current non-cyclical pain | M (SD); N<br>Mdn (IQR) | 55.29 (34.86), 7<br>37.00 (24.00 – 92.00) | 52.40 (30.25), 267<br>56.00 (27.00 – 77.00) | 0.776[2] |
| Current dyspareunia | M (SD); N<br>Mdn (IQR) | 35.33 (43.56), 3<br>22.00 (0.00 – 84.00) | 44.14 (35.43), 254<br>45.00 (8.00 – 73.00) | 0.611[2] |
| Current dysuria | M (SD); N<br>Mdn (IQR) | 32.83 (35.63), 6<br>28.00 (1.00 – 50.00) | 29.09 (30.93), 250<br>16.00 (2.00 – 51.00) | 0.856[2] |
| Current dyschezia | M (SD); N<br>Mdn (IQR) | 65.33 (25.49), 6<br>70.00 (48.00 – 87.00) | 39.56 (32.31), 255<br>36.00 (9.00 – 66.00) | 0.054[2] |
| Current lower back pain | M (SD); N<br>Mdn (IQR) | 44.50 (33.09), 6<br>41.50 (19.00 – 75.00) | 58.71 (34.09), 265<br>64.00 (28.00 – 88.00) | 0.270[2] |
| Current global pain | M (SD); N<br>Mdn (IQR) | 49.67 (24.04), 2<br>49.67 (32.67 – 66.67) | 46.51 (21.37), 223<br>45.83 (30.17 – 62.33) | 0.827[2] |
| Pain-induced disability | | | | |
| Family prior to i/q | M (SD); N<br>Mdn (IQR) | 5.84 (2.35), 37<br>6.00 (5.00 – 8.00) | 5.11 (2.48), 277<br>5.00 (3.00 – 7.00) | 0.095[2] |
| Recreational prior to i/q | M (SD); N<br>Mdn (IQR) | 5.84 (2.59), 37<br>7.00 (4.00 – 8.00) | 5.56 (2.56), 277<br>6.00 (4.00 – 8.00) | 0.468[2] |
| Social activities prior to i/q | M (SD); N<br>Mdn (IQR) | 5.95 (2.81), 37<br>6.00 (4.00 – 8.00) | 5.41 (2.73), 277<br>6.00 (3.00 – 8.00) | 0.237[2] |
| Occupational prior to i/q | M (SD); N<br>Mdn (IQR) | 6.54 (2.56), 37<br>7.00 (4.00 – 8.00) | 5.96 (2.81), 277<br>6.00 (4.00 – 8.00) | 0.286[2] |
| Sexuality prior to i/q | M (SD); N<br>Mdn (IQR) | 5.77 (3.44), 35<br>6.00 (3.00 – 9.00) | 6.05 (3.24), 274<br>7.00 (3.00 – 9.00) | 0.692[2] |
| Self-care prior to i/q | M (SD); N<br>Mdn (IQR) | 2.70 (3.19), 37<br>1.00 (0.00 – 5.00) | 2.74 (2.77), 277<br>2.00 (0.00 – 5.00) | 0.562[2] |
| Life support prior to i/q | M (SD); N<br>Mdn (IQR) | 2.73 (2.88), 37<br>2.00 (0.00 – 5.00) | 2.67 (2.62), 277<br>2.00 (0.00 – 5.00) | 0.841[2] |
| Discretional activities prior to i/q | M (SD); N<br>Mdn (IQR) | 29.80 (11.56), 35<br>31.00 (23.00 – 38.00) | 28.07 (11.06), 274<br>29.50 (11.06 – 37.00) | 0.340[2] |
| Basic activities prior to i/q | M (SD)<br>Mdn (IQR) | 5.43 (5.64), 35<br>4.00 (0.00 – 9.00) | 5.42 (4.85), 274<br>4.00 (1.00 – 9.00) | 0.628[2] |
| Global PDI prior to i/q | M (SD); N<br>Mdn (IQR) | 35.23 (15.64), 35<br>36.00 (26.00 – 46.00) | 33.4 (14.19), 274<br>34.00 (23.00 – 43.00) | 0.560[2] |
| Current family activities | M (SD); N<br>Mdn (IQR) | 4.00 (3.16), 5<br>4.00 (2.00 – 6.00) | 5.33 (2.69), 277<br>5.00 (3.00 – 8.00) | 0.321[2] |
| Current recreational activities | M (SD); N<br>Mdn (IQR) | 3.80 (3.03), 5<br>3.00 (1.00 – 7.00) | 5.34 (2.90), 277<br>6.00 (3.00 – 8.00) | 0.278[2] |
| Current social activities | M (SD); N<br>Mdn (IQR) | 1.75 (2.87), 4<br>0.50 (0.00 – 3.50) | 4.53 (3.43), 277<br>5.00 (1.00 – 8.00) | 0.112[2] |
| Current occupational activities | M (SD); N<br>Mdn (IQR) | 3.25 (3.59), 4<br>2.50 (0.50 – 6.00) | 5.30 (3.26), 277<br>5.00 (3.00 – 8.00) | 0.216[2] |
| Current sexuality | M (SD); N<br>Mdn (IQR) | 0.00 (0.00), 1<br>0.00 (0.00 – 0.00) | 5.54 (3.48), 277<br>6.00 (3.00 – 9.00) | 0.144[2] |

| | | | | |
|---|---|---|---|---|
| Current self-care | M (SD); N<br>Mdn (IQR) | 0.40 (0.89), 5<br>0.00 (0.00 – 0.00) | 2.87 (2.84), 277<br>2.00 (0.00 – 5.00) | **0.025**[2] |
| Current life support | M (SD); N<br>Mdn (IQR) | 1.20 (1.64), 5<br>1.00 (0.00 – 1.00) | 2.69 (2.79), 277<br>2.00 (0.00 – 5.00) | 0.282[2] |
| Current discretional activities | M (SD); N<br>Mdn (IQR) | n.a.<br>n.a. | 26.05 (12.54), 277<br>26.00 (16.00 – 36.00) | n.a. |
| Current basic activities | M (SD); N<br>Mdn (IQR) | n.a.<br>n.a. | 5.56 (5.12), 277<br>5.00 (1.00 – 9.00) | n.a. |
| Current global PDI | M (SD); N<br>Mdn (IQR) | n.a.<br>n.a. | 31.61 (15.82); 277<br>32.00 (19.00 – 43.00) | n.a. |
| Mental outcomes | | | | |
| PHQ-2 | M (SD); N<br>Mdn (IQR) | 2.00 (2.35); 5<br>1.00 (1.00 – 2.00) | 2.84 (1.68); 269<br>2.00 (2.00 – 4.00) | 0.163[2] |
| GAD-2 | M (SD); N<br>Mdn (IQR) | 1.60 (1.52); 5<br>1.00 (1.00 – 2.00) | 2.91 (1.83); 269<br>2.00 (2.00 – 4.00) | 0.092[2] |
| PHQ-4 | M (SD); N<br>Mdn (IQR) | 3.60 (3.85); 5<br>2.00 (2.00 – 4.00) | 5.75 (3.19); 269<br>5.00 (3.00 – 8.00) | 0.102[2] |
| BRS | M (SD); N<br>Mdn (IQR) | 2.30 (1.16); 6<br>2.25 (1.33 – 3.33) | 2.76 (0.82); 267<br>2.66 (2.16 – 3.33) | 0.338[2] |

PDI = Pain Disability Index; BRS = Brief Resilience Scale; GAD-2 = Generalized Anxiety Disorder Scale; PHQ-2 = Patient Health Questionnaire for Depression; PHQ-4 = Patient Health Questionnaire for Depression and Anxiety; i/q =isolation or quarantine; d = days; N = Number of women for which data were available; n = sample size; M = mean; SD = standard deviation, Mdn = median; IQR = Interquartile Range; n.a.= not available/not applicable; y = years. Values in bold indicate statistical significance, as the level of statistical significance was set to $p<0.05$ ([1] = $\chi^2$-test; [2] = Mann-Whitney-U-test; [3] = Fisher exact test).
